# Time-dependent recurrence and resolution of pigment epithelial detachment in central serous chorioretinopathy

**DOI:** 10.1186/s12886-023-02882-9

**Published:** 2023-04-03

**Authors:** Yang Meng, Liao Chen, Lu Li, Yishuang Xu, Yu Su, Lu Zhang, Zuohuizi Yi, Changzheng Chen

**Affiliations:** 1 Department of Ophthalmology, Renmin Hospital of Wuhan University, 238 Jiefang Road, Wuhan, China; 2 Department of Ultrasound Imaging, Renmin Hospital of Wuhan University, 238 Jiefang Road, Wuhan, China; 3 Department of Ophthalmology, The Central Hospital of Wuhan, 26 Shengli Road, Wuhan, China

**Keywords:** Retinal pigment epithelium, Pigment epithelial detachment, Central serous chorioretinopathy, Glucocorticoid, Cortisol

## Abstract

**Background:**

Cortisol plays a role in the pathogenesis of central serous chorioretinopathy (CSC). CSC patients have abnormal time-dependent changes in cortisol levels. Here we report a rare case of a patient with central serous chorioretinopathy whose pigment epithelial detachment (PED) exhibited time-dependent recurrence and resolution.

**Case presentation:**

A 47-year-old man presented in 2016 for vision loss in the left eye related to recurrent CSC. During follow-up, his PED was observed to resolve spontaneously while he was still in our clinic and recurred the next morning. Such time-dependent changes of the PED were observed in several next follow-ups without any intervention. After excluding possible external factors, the abnormal diurnal variation of cortisol was considered as the internal factor affecting PED.

**Conclusions:**

This is the first article that described the spontaneous time-dependent recurrence and resolution of PED without external interference, where endogenous cortisol may be responsible. Interventions against the abnormal cortisol level might be a potential treatment strategy for CSC. More research is urged to explore the impact of the diurnal change in cortisol levels on eyes with CSC.

## Background

Central serous chorioretinopathy (CSC), first described by Albrecht von Graefe in 1866 as recurrent central retinitis, is characterized by serous detachment of the neurosensory retina with or without pigment epithelial detachment (PED) [1–3]. Although 157 years have passed since its first description, the exact etiology of CSC is still eluding ophthalmologists.

Hormonal dysregulation plays an important role in the pathogenesis of CSC, especially cortisol, the predominant endogenous glucocorticoid in humans [4–8]. The hypothalamus–pituitary–adrenal (HPA) axis is an important physiological response system that is usually activated when the body is in an acutely stressful situation [9]. Cortisol is the end product of the HPA axis and plays a central role in the body’s stress response [10]. A previous study showed that CSC patients had higher cortisol levels in the morning than healthy controls, but the difference disappeared in the evening [11]. From this perspective, it is reasonable that abnormal time-dependent changes in cortisol levels may lead to dynamic changes in the eyes with CSC (e.g. subretinal fluid or PED). However, the potential effect of this time-dependent abnormal change in cortisol levels on CSC has not been directly observed yet.

Here, we report a rare and interesting case of a man with CSC whose PED was always found to recur in the morning and resolve before the afternoon, which was found several times during his follow-up visits to our clinic.

## Case presentation

On May 10, 2016, a 47-year-old man was referred to our clinic for recurrent vision loss and relative central scotoma in the left eye. He had had two previous CSC episodes in 2015. Any other medical history or drug use was denied. Visual acuity was 20/20 in his right eye and 20/50 in his left eye.

**Fig. 1:**
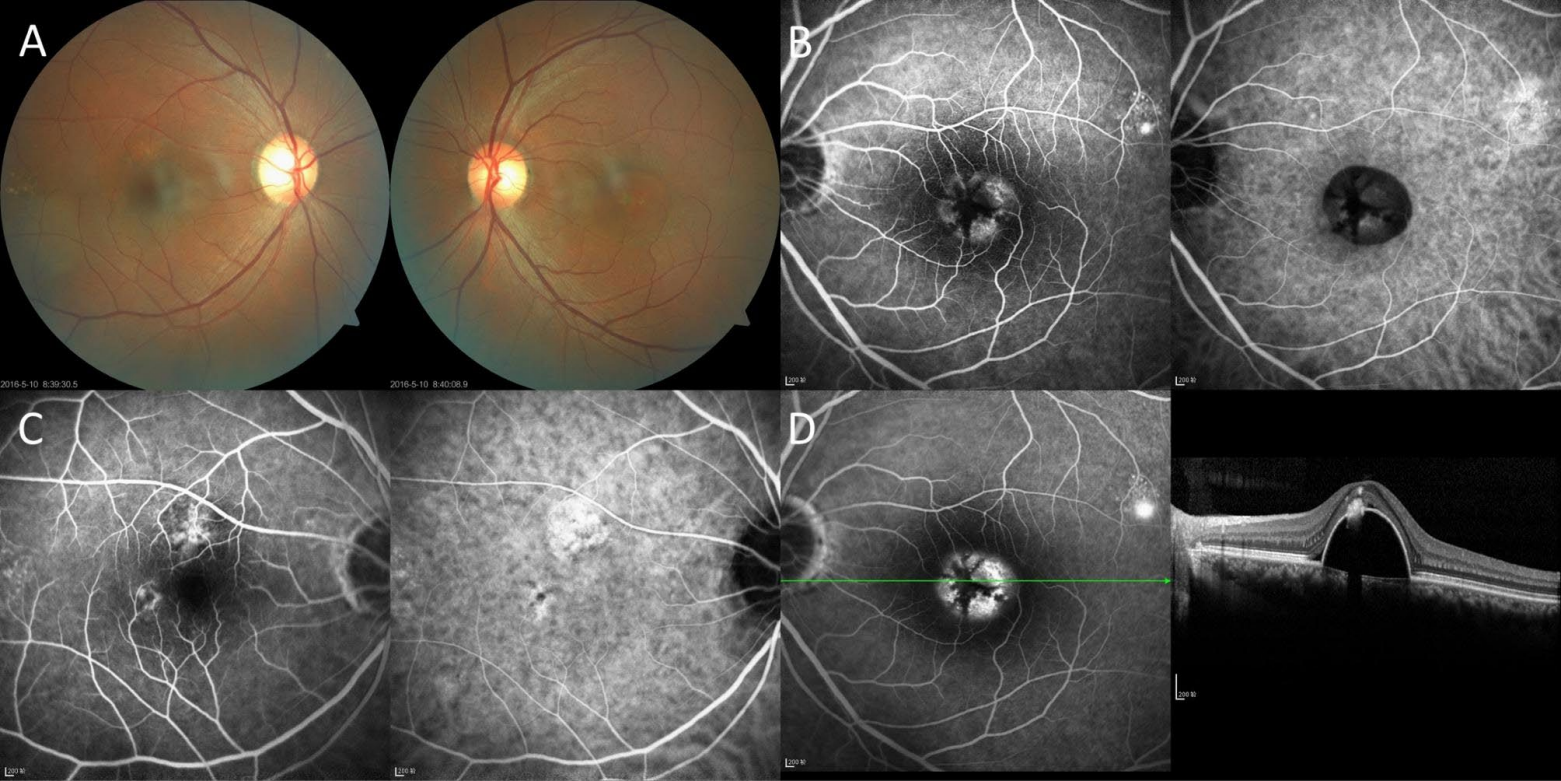
CFP, FFA, ICGA, and OCT examinations of the patient at presentation **A**: CFP showed some hyperpigmented RPE lesions at the posterior pole of the right eye as well as a clear-edged round-like area of retinal elevation in the macula of the left eye. **B**: FFA and ICGA at 2 min of the left eye. FFA showed a round-like dye pooling with radial blocking in the macula and a leakage point in the supratemporal side of the macula. ICGA revealed a well-defined round-like hypofluorescence with radial blocking inside and a patchy area of hyperfluorescence in the supratemporal side of the macula. **C**: FFA and ICGA at 2 min of the right eye. FFA showed 2 patchy hyperfluorescent areas around the macula. ICGA demonstrated a patchy hyperfluorescence on the upper side of the macula, and a small hypofluorescent dot with surrounding hyperfluorescence on the inferotemporal side of the macula. **D**: FFA at 8 min and a horizontal OCT scan across the macula of the left eye. The dye pooling and the leakage point became brighter than 2 min. OCT demonstrated the presence of a dome-shaped PED with a hyperreflective material on the top and minimal serous detachment of the neurosensory retina.

Intraocular pressure, eye movements, and anterior segment examination were unremarkable in both eyes. Dilated funduscopic examination demonstrated some hyperpigmented retinal pigment epithelium (RPE) lesions in the right eye and retinal elevation in the macular region of the left eye (Fig. 1A). The patient was diagnosed with CSC recurrence in the left eye and pachychoroid pigment epitheliopathy (PPE) in the right eye after receiving color fundus photography (CFP), fundus fluorescein angiography (FFA), indocyanine green angiography (ICGA), and optical coherence tomography (OCT) examinations (Fig. 1B-D). The patient chose to receive photodynamic therapy (PDT) treatment in a local hospital.

**Fig. 2:**
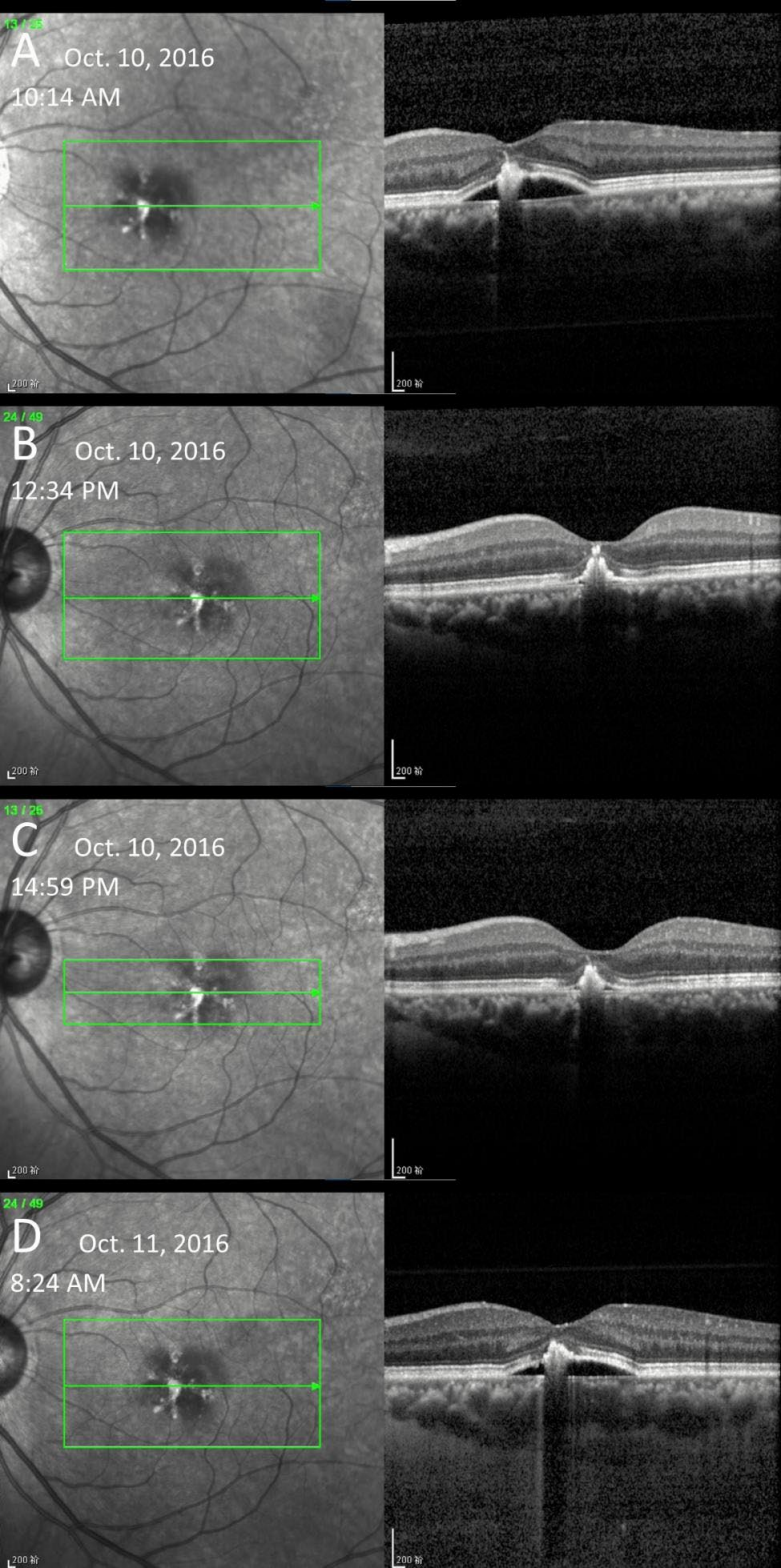
OCT images of the patient at the first follow-up **A**: The PED existed in the morning. **B**: At noon, the PED had spontaneously subsided. **C**: OCT image in the afternoon confirmed the resolution of PED again. **D**: The PED recurred the next morning.

On November 10, 2016, he presented again for an assessment of the left eye. Visual acuity was 20/20 in the right eye and 10/20 in the left eye. His right eye remained stable while CFP, FFA, ICGA, and OCT examinations confirmed CSC recurrence in the left eye. However, the PED was resolved spontaneously while he was still in our clinic and recurred the next morning (Fig. 2). The OCT image taken on his arrival at our clinic showed the existence of a PED, which was flatter than 5 months ago (Fig. 2A). However, about two and a half hours later, a second OCT scan showed the absence of his PED (Fig. 2B). A third OCT scan taken about 5 h later in the afternoon definitively confirmed the resolution of the PED again (Fig. 2C). The patient was asked to return to the clinic the next morning. To our surprise, the OCT image showed the recurrence of PED (Fig. 2D). This time, the patient was asked to receive regular follow-ups to monitor the disease progression.

The same situation was observed again on December 1, 2016, that is, the PED appeared in the morning of the first day, was completely absorbed in the afternoon, and recurred the next morning. This time dilated FFA or ICGA examinations were not conducted so the influence of contrast agents (sodium fluorescein and indocyanine green) and mydriatic drugs can be excluded.

The pattern of spontaneous resolution of PED within the day was continuously observed in several next follow-ups. During this period, we have tried many ways to explore the possible external factors that may affect the PED. We tried to use the single-line scanning mode to image the PED quickly instead of using the time-consuming multi-line scanning mode, so as to eliminate the possible impact of long-time OCT or infrared (IR) imaging on PED. Besides, the patient was required to sit still in a comfortable chair during the examinations to avoid the possible impact of physical activity and nervousness on his PED. He was also asked to avoid consuming tea, coffee, chocolate, cola, and other foods that might excite him two days before and on the day of the examination. However, all these changes did not accelerate or slow down the resolution of PED.

**Fig. 3:**
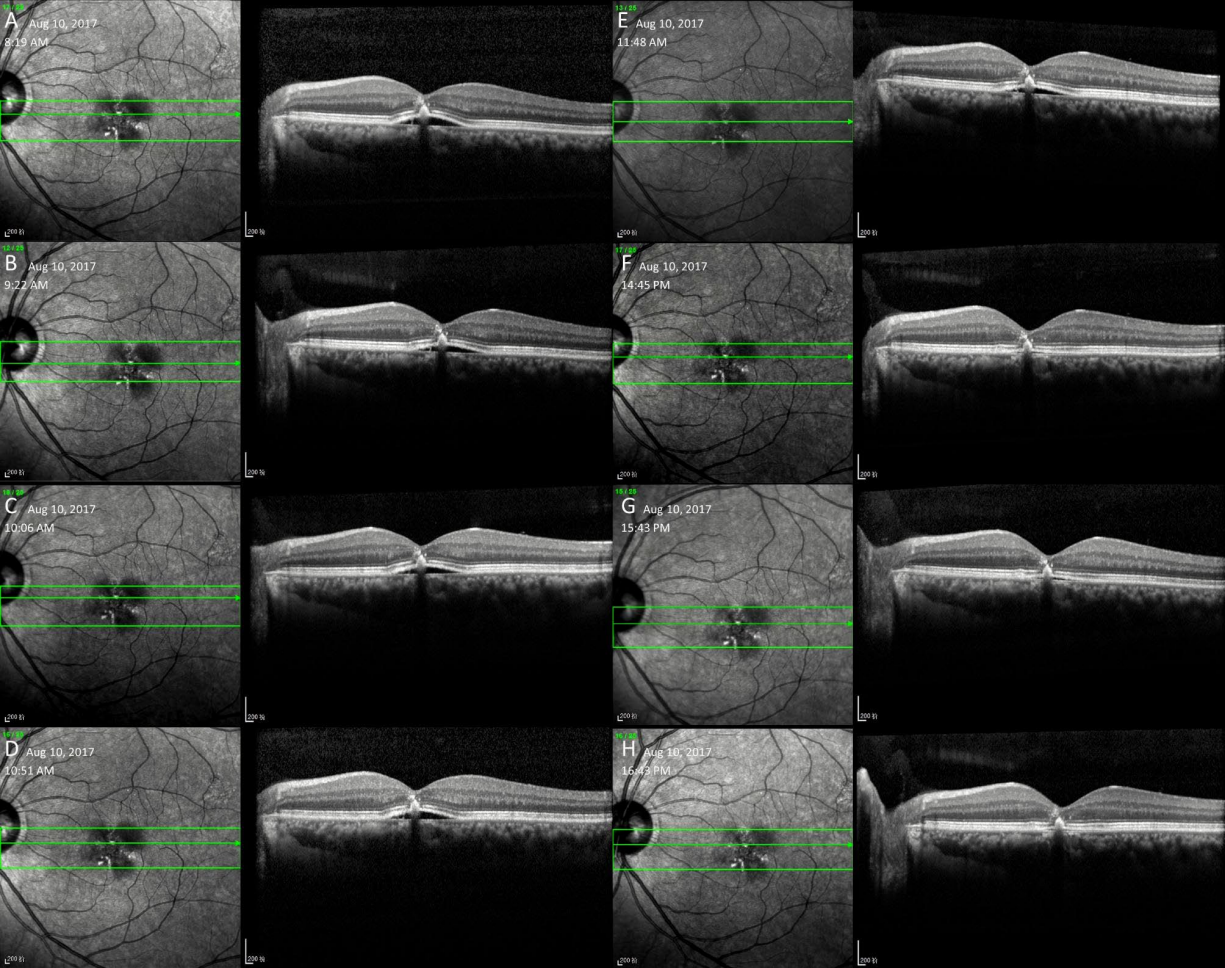
OCT images of the patient when spontaneous resolution of PED was last observed **A**: The PED existed in the morning. **B**-**D**: The PED remained generally stable. **E**: Around noon, the PED still existed but became much shallower. **F**: The PED basically disappeared, leaving only a very small amount of sub-RPE fluid. **H**: The PED disappeared completely.

**Fig. 4:**
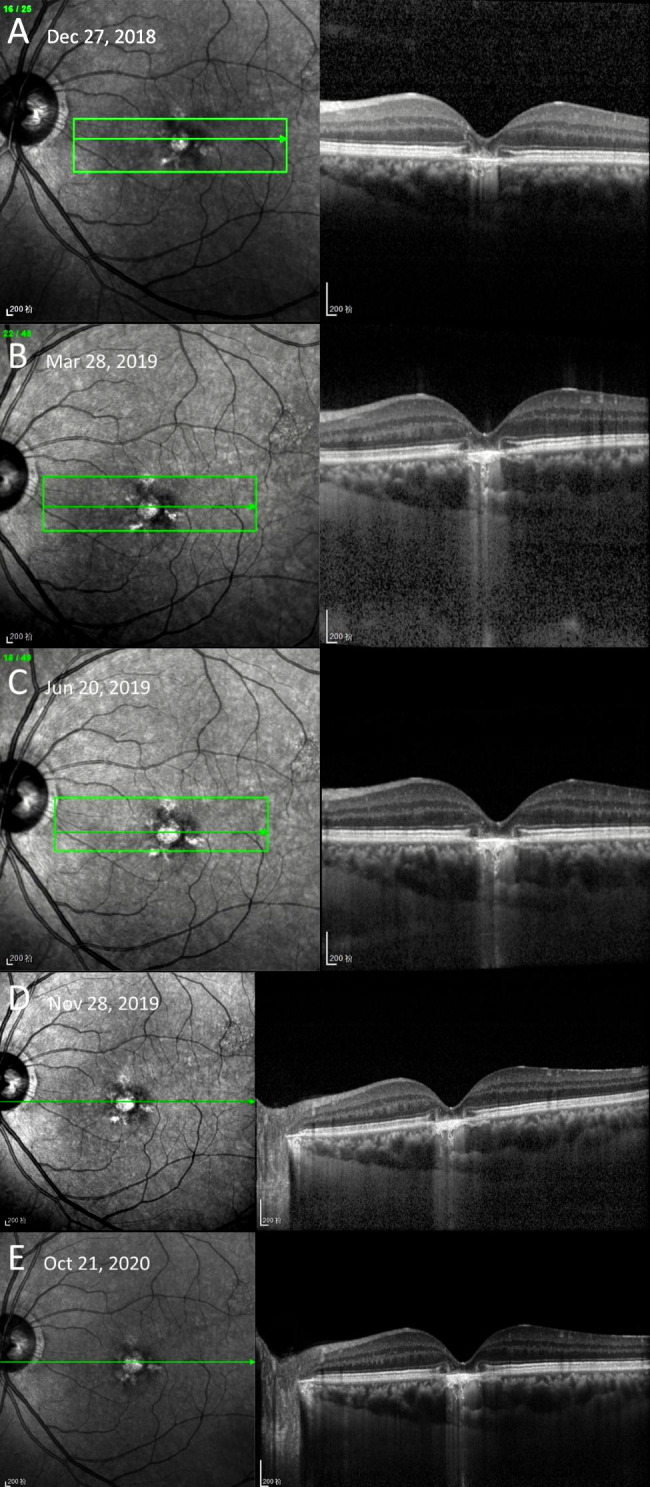
OCT images of the sub-foveal RPE atrophy **A**-**E**: The RPE atrophy slightly expanded during the last two years of follow-up. Dilated choroidal vessels could be seen at each follow-up.

His last follow-up to show the spontaneous resolution pattern of PED was on August 10, 2017 (Fig. 3). Consistent with previous follow-ups, this time the PED also appeared in the morning and disappeared in the afternoon. One year later, when the patient came back, visual acuity dropped to 20/100 in the left eye. OCT scans showed that there was sub-foveal RPE atrophy instead of a PED (Fig. 4A). In the following two years, the RPE atrophy slightly enlarged, and the patient’s visual acuity eventually decreased to 20/200 at his most recent visit (Fig. 4B-E**)**. From the discovery of time-dependent recurrence and resolution of his PED to his last follow-up, his right eye kept a visual acuity of 20/20. The patient was a non‑smoker, and no drugs were used during the follow-ups. The patient had good sleep quality and a well-regulated lifestyle. No other concomitant diseases were found despite general and ophthalmic examinations.

## Discussion and conclusions

PED is an important feature of CSC with vision-threatening potential and is reported to occur in 9%~100% of CSC cases [4]. To date, the exact pathophysiology of both CSC and PED still remains enigmatic.

Current theories have put CSC as a pachychoroid spectrum disease and confirmed the role of choroidal changes and RPE defects in the pathogenesis of CSC, with increasing literature paying attention to the role of hormonal dysregulation in the meantime [4]. Glucocorticoids, both exogenous and endogenous, have been reported to have an association with CSC [5, 6]. By far, the most widely studied hormone in CSC is cortisol, the predominant endogenous glucocorticoid in humans [6–8, 11]. In a meta-analysis, the CSC group had much higher serum cortisol levels than the non-CSC group, and there was a significant association between serum cortisol levels and the risk of CSC [6]. Scarinci et al. found that CSC patients had statistically higher salivary cortisol concentrations at awakening (around 7 a.m.) and at 30 and 60 min after awakening than healthy controls, indicating that CSC patients had a stronger cortisol awakening response [11]. Then, the difference narrowed steadily throughout the day and disappeared before 8 p.m. [11].

CSC after the use of exogenous glucocorticoids has been widely reported in the literature, including oral, inhaled, intranasal, intravenous, intramuscular, intraarticular, and periocular administrations, etc. [2, 5, 12–15]. Prakash et al. have reported a case where a 35-year-old male experienced four recurrent episodes of CSC within one year, a few days before each episode he was using dexamethasone eye drops intranasally for rhinitis [14]. Fardin et al. reported a case of a 40-year-old female who developed CSC after starting to use oral inhaled fluticasone for post-mycoplasmal bronchospasm, and her CSC resolved after cessation of glucocorticoid inhalation and did not relapse within the next two years [15].

It is well known that the dose of inhaled glucocorticoids or glucocorticoid drops is quite low when compared to oral or intravenous glucocorticoids. Now that low-dose exogenous glucocorticoids can lead to CSC, then the abnormal fluctuations of endogenous glucocorticoids (cortisol) in the body may also lead to the occurrence or recurrences of CSC. Glucocorticoids may directly damage the RPE cells or their tight junctions, thus letting fluid from choroidal vessels accumulate under the retina, resulting in CSC [6, 16]. Glucocorticoids can also up-regulate the endothelial vasodilatory calcium-dependent potassium channel KCa2.3, causing choroidal vascular dilation in CSC [4]. It is also noticed that glucocorticoids can suppress collagen synthesis, a crucial component of the Bruch’s membrane, promoting persistent focal leakage from choroidal vessels into subretinal space [17]. In short, glucocorticoids may promote the development of CSC in various ways.

As aforementioned, the difference in cortisol concentration between CSC patients and healthy controls is the biggest in the morning, narrowed throughout the day, and finally disappeared before evening [11]. Usually, cortisol levels follow a “diurnal variation” pattern. Cortisol concentration increases in the second half of the night, peaks in the early morning, declines throughout the day, and reaches the lowest level in the first half of the night [10]. Based on the findings of Scarinci et al., we can reasonably speculate that the cortisol level of CSC patients increases much more than healthy people during the night [11].

The above may explain the time-dependent recurrence and resolution of PED observed in our case. Specifically, his higher cortisol level above basal in the morning could lead to the recurrence of PED. As the cortisol level kept approaching normal, his PED began to resolve. The next morning, the PED appeared again due to the diurnal variation of cortisol. A similar situation is that pregnancy is considered to trigger CSC, which is the main cause of acquired visual impairment in pregnant women whereas increased endogenous glucocorticoid during pregnancy is thought to contribute to CSC [4, 18]. It should be noted that the pathological mechanism of CSC is complex and has not been well understood. Although we have excluded relevant external influencing factors as much as possible, the time-dependent changes of PED observed by us may not be the result under the single influence of the fluctuation of cortisol level. Still, we provide a rare and enlightening case, which is helpful to enrich our understanding of CSC to a certain extent.

In summary, this paper, for the first time, provided a rare case of a man with CSC whose PED followed a pattern of time-dependent recurrence and resolution for a period. The PED always appeared in the morning and disappeared in the afternoon, which is likely due to the abnormal diurnal variation of cortisol after excluding possible external factors. Interventions against the abnormal cortisol level might be a potential treatment strategy for CSC patients. More research is needed to explore the impact of the diurnal change in cortisol levels on eyes with CSC.

## Data Availability

All data and materials supporting our findings are contained within this manuscript.

## Abbreviations

CSC: central serous chorioretinopathy
PED: pigment epithelial detachment
HPA: hypothalamus–pituitary–adrenal
RPE: retinal pigment epithelium
PPE: pachychoroid pigment epitheliopathy
CFP: color fundus photography
FFA: fundus fluorescein angiography
ICGA: indocyanine green angiography
OCT: optical coherence tomography
PDT: photodynamic therapy
IR: infrared.

## References

[1] Albrecht von Graefe (1866). Ueber zentrale recidivierende Retinitis. Graefes Arch Clin Exp Ophthalmol.

[2] Chronopoulos A, Kakkassery V, Strobel MA (2021). The significance of pigment epithelial detachment in central serous chorioretinopathy. Eur J Ophthalmol.

[3] Daruich A, Matet A, Dirani A (2015). Central serous chorioretinopathy: recent findings and new physiopathology hypothesis. Prog Retin Eye Res.

[4] Kaye R, Chandra S, Sheth J (2020). Central serous chorioretinopathy: an update on risk factors, pathophysiology and imaging modalities. Prog Retin Eye Res.

[5] Nicholson BP, Atchison E, Idris AA, Bakri SJ (2018). Central serous chorioretinopathy and glucocorticoids: an update on evidence for association. Surv Ophthalmol.

[6] Liang ZQ, Huang LZ, Qu JF, Zhao MW (2018). Association between endogenous cortisol level and the risk of central serous chorioretinopathy: a Meta-analysis. Int J Ophthalmol.

[7] Schellevis RL, Altay L, Kalisingh A (2019). Elevated steroid hormone levels in active chronic Central Serous Chorioretinopathy. Invest Ophthalmol Vis Sci.

[8] Tufan HA, Gencer B, Comez AT (2013). Serum cortisol and testosterone levels in chronic central serous chorioretinopathy. Graefes Arch Clin Exp Ophthalmol.

[9] Fogelman N, Canli T (2018). Early life stress and cortisol: a meta-analysis. Horm Behav.

[10] Fries E, Dettenborn L, Kirschbaum C (2009). The cortisol awakening response (CAR): facts and future directions. Int J Psychophysiol.

[11] Scarinci F, Patacchioli FR, Palmery M (2020). Diurnal trajectories of salivary cortisol and α-amylase and psychological profiles in patients with central serous chorioretinopathy. Chronobiol Int.

[12] Wakakura M, Ishikawa S (1984). Central serous chorioretinopathy complicating systemic corticosteroid treatment. Br J Ophthalmol.

[13] Carvalho-Recchia CA, Yannuzzi LA, Negrão S (2002). Corticosteroids and central serous chorioretinopathy. Ophthalmology.

[14] Prakash G, Shephali J, Tirupati N, Ji PD (2013). Recurrent Central Serous Chorioretinopathy with Dexamethasone Eye Drop used Nasally for Rhinitis. Middle East Afr J Ophthalmol.

[15] Fardin B, Weissgold DJ (2002). Central serous chorioretinopathy after inhaled steroid use for post-mycoplasmal bronchospasm. Br J Ophthalmol.

[16] Cusani M (2004). Central serous chorioretinopathy and glucocorticoids. Surv Ophthalmol.

[17] Norouzpour A, Abrishami M (2016). Central serous chorioretinopathy: from glucocorticoids to light intensity. Int J Ophthalmol.

[18] Pofi R, Tomlinson JW (2020). Glucocorticoids in pregnancy. Obstet Med.

